# Molecular Studies for the Early Detection of Philadelphia-Negative Myeloproliferative Neoplasms

**DOI:** 10.3390/ijms241612700

**Published:** 2023-08-11

**Authors:** Ruth Stuckey, Cristina Bilbao-Sieyro, Adrián Segura-Díaz, María Teresa Gómez-Casares

**Affiliations:** 1 Hematology Department, Hospital Universitario de Gran Canaria Dr. Negrín, 35019 Las Palmas de Gran Canaria, Spain; rstuckey@fciisc.es (R.S.); bilbaocristina@gmail.com (C.B.-S.); asegdia@gobiernodecanarias.org (A.S.-D.); 2 Morphology Department, Universidad de Las Palmas de Gran Canaria, 35016 Las Palmas de Gran Canaria, Spain; 3 Department of Medical Sciences, Universidad de Las Palmas de Gran Canaria, 35016 Las Palmas de Gran Canaria, Spain

**Keywords:** Philadelphia-negative myeloproliferative neoplasms, clonal hematopoiesis of indeterminate potential (CHIP), clonal expansion, screening, fitness, thrombosis, intervention

## Abstract

*JAK2* V617F is the predominant driver mutation in patients with Philadelphia-negative myeloproliferative neoplasms (MPN). *JAK2* mutations are also frequent in clonal hematopoiesis of indeterminate potential (CHIP) in otherwise “healthy” individuals. However, the period between mutation acquisition and MPN diagnosis (known as latency) varies widely between individuals, with *JAK2* mutations detectable several decades before diagnosis and even from birth in some individuals. Here, we will review the current evidence on the biological factors, such as additional mutations and chronic inflammation, which influence clonal expansion and may determine why some *JAK2*-mutated individuals will progress to an overt neoplasm during their lifetime while others will not. We will also introduce several germline variants that predispose individuals to CHIP (as well as MPN) identified from genome-wide association studies. Finally, we will explore possible mutation screening or interventions that could help to minimize MPN-associated cardiovascular complications or even delay malignant progression.

## 1. Introduction

The Philadelphia-negative myeloproliferative neoplasms (MPN) are a group of clonal diseases that affect the hematopoeitic stem and progenitor cells (HSPC) and are characterized by the abnormal proliferation of one or more myeloid cell lines [1]. The “classic” MPNs, which include polycythemia vera (PV), essential thrombocythemia (ET), and myelofibrosis (MF), share a common pathogenic mechanism: the aberrant activation of the JAK/STAT signaling pathway. This pathway is responsible for the regulation of cellular proliferation, differentiation, and apoptosis [2].

The most frequent mechanism of JAK/STAT activation in MPNs is the point mutation *JAK2* p.V617F, which is found in 97% of PV patients, 50–60% of ET patients, and 55–60% of MF patients [3], although the variant allele frequency (VAF) is generally lower for ET patients than PV or MF patients [4,5,6]. This gain-of-function mutation has been shown to be clonal [7,8,9] and is present in HSPCs [10]. Since 2016, the presence of mutations in the driver genes *JAK2*, *CALR*, and *MPL* has been considered a major diagnostic criterion in MF and ET by the World Health Organization. In PV, the major diagnostic criterion is the detection of *JAK2* p.V617F or mutations in exon 12 of *JAK2* [1,11].

Common MPN-related complications include thrombosis (arterial, venous, and of the microcirculation) and hemorrhage, which are major causes of morbidity and mortality in MPN patients. Thrombotic events frequently occur at diagnosis or several years before diagnosis [12], even in young patients [13]. Additionally, patients with ET and PV may transform to MF, and some patients with MPN will progress to acute leukemia, with an estimated incidence at 10 years ranging from approximately 1% of patients with ET to 10–20% of those with MF [14,15].

In cancer, the primary concept of early diagnosis is to detect “symptomatic patients as early as possible”, while screening “consists of testing healthy individuals to identify those having cancers before any symptoms appear” [16]. Both early diagnosis and screening can help prevent the development of later complications. The National Cancer Institute’s “PreCancer Atlas” is an innovative initiative currently underway to elucidate the early changes associated with premalignancy and how cancers develop, with the monumental objective of applying this knowledge to inform the development of early detection strategies, preventative measures and potential new treatments for individuals with pre-cancer [17].

The observation that *JAK2*-mutated clones generally increase in VAF over time suggests that monitoring the VAF of *JAK2* p.V617F could be useful for detecting the progression from an early-phase MPN to an overt MPN [18]. The concept of screening refers to the use of simple tests across a healthy population to identify those individuals who have a disease but do not yet have symptoms. At face value, identifying patients with early-phase MPN may seem straightforward: screening for the *JAK2* p.V617F mutation before other disease-related signs appear, such as changes in the blood count. However, molecular diagnosis based solely on the detection of *JAK2* p.V617F is not specific for classic MPNs. This variant can also be found in other myeloid neoplasms, including acute myeloid leukemia (AML), myelodysplastic syndrome (MDS), chronic myelomonocytic leukemia (CMML), and systemic mastocytosis [19,20]. Moreover, in 2006 the variant was identified for the first time in 5 healthy individuals out of 52 tested (9.6%), albeit with a low VAF [21].

## 2. Clonal Hematopoiesis of Indeterminate Potential (CHIP)

Across the lifespan of every individual, our cells accumulate mutations (or other genetic alterations) with each cell cycle. Thus, the frequency of somatic mutations increases linearly with age, estimated at rates of 0.05 × 10^−9^ mutations per nucleotide per cell division [22]. These mutations may be deleterious and cause cell death, be neutral, or confer a competitive advantage to the cell, resulting in the expansion of a new clone, increasing cell “fitness” [23,24]. As such, age and cancer development are undoubtedly related [25].

For HSPC of the bone marrow (BM), which divide every 2 to 20 months, estimates for the introduction of mutations vary from one to two mutations per cell division to just 14 mutations per year [26,27]. In this way, if the genetic alterations affect genes important in hematopoiesis, they may provide a selective advantage to the cell, resulting in clonal hematopoiesis [27,28]. *JAK2* p.V617F and other gain-of-function variants that result in the constitutive activation of JAK2’s kinase activity are known to confer HSPCs with a survival advantage [29].

Clonal hematopoiesis of indeterminate potential (CHIP) refers to a population of cells harboring somatic mutations that confer a fitness advantage and are detected in the blood of individuals without evidence of a hematological anomaly or cytopenias. In addition to somatic mutations, CHIP may also be a result of translocations and copy number alterations (CNA), such as the loss of chromosome Y or deletions of chromosomes 20q and 13q [30,31]. Recently, Niroula et al. described the existence of lymphoid CHIP (L-CHIP), in addition to the commonly described myeloid CHIP (M-CHIP), in healthy individuals. The presence of L-CHIP was associated with lymphoid neoplasms, while M-CHIP was associated with myeloid neoplasms [32].

In early studies, the incidence of CHIP was shown to increase with age, with a prevalence of M-CHIP of 1% in those aged under 50 years, reaching 10% to 15% in those over 70 years (with a prevalence of L-CHIP somewhat lower) [33]. The detection of CHIP, including somatic mutations and/or chromosomal alterations, may be significant as it has been associated with a higher risk of developing myeloid and lymphoid neoplasms [32]. In fact, M-CHIP was included in the new World Health Organization (WHO) classification of myeloid neoplasms due to its recognition as a myeloid precursor lesion [1]. Furthermore, M-CHIP is associated with a 40% higher mortality due to an increased risk of developing cardiovascular diseases [33,34,35]. However, unlike M-CHIP, the presence of L-CHIP was not associated with increased cardiovascular risk or mortality rate [32].

Approximately half of all MPN patients harbor mutations in the CHIP genes *TET2*, *DNMT3A*, and *ASXL1* [33,36,37]. Additionally, the occurrence of CHIP mutations in *JAK2* in the healthy population, including the *JAK2* p.V617F variant, is quite frequent. For instance, one 2007 study detected *JAK2* p.V617F in 0.94% (37/3935) of randomly analyzed peripheral blood (PB) samples in a routine laboratory setting [38]. Another study from 2019 utilized digital PCR to screen a healthy Danish population cohort and found mutations in *JAK2* and *CALR* in 3.1% (613/19,958) and 0.16% (32/19,958) of the samples, respectively [39].

Is the Acquisition of the JAK2 p.V617F Variant Sufficient for the Development of an MPN?

Experiments conducted on mice whose BM was transduced with a retrovirus expressing *JAK2* p.V617F, demonstrated that expression of the variant alone was sufficient to induce an MPN phenotype [40,41]. Nevertheless, the MPN observed in these mice was polyclonal in nature, unlike the monoclonal MPN seen in patients. Lundberg et al. observed that an MPN phenotype could be initiated in some mice with the transplantation of a single cell with the *JAK2* p.V617F variant, but not in all mice [42]. Similarly, in a Danish cohort of healthy individuals, the development of MPN was confirmed in only 14 out of 613 individuals (2.3%) with *JAK2* mutations and 2 out of 32 (6.3%) individuals with *CALR* mutations [39]. Taken together, these experiments suggest that only a small proportion of individuals with a *JAK2* mutation will develop an MPN in their lifetime.

So, what determines the evolution of progression of an MPN after the acquisition of a *JAK2* mutation or other driver mutation? Might all individuals with CHIP be expected to develop an overt MPN if they lived long enough?

## 3. Variant Allele Frequency

In patients with a confirmed MPN diagnosis, the *JAK2* p.V617F variant can be detected in peripheral blood at a VAF of 1% [43] or below using quantitative PCR [44,45,46], and with a detection limit of 0.01% using digital PCR [39,47]. While quantification of the VAF is not obligatory for the diagnosis or monitoring of MPN patients during follow-up, various studies have associated a higher *JAK2* p.V617F VAF with a greater symptom burden [48,49,50,51], as well as an increased risk of thrombosis [52,53] and fibrotic progression [49,54,55]. It has also been demonstrated that a larger clone size, as indicated by a higher VAF, is associated with a higher risk of developing a hematological neoplasm. For example, Jaiswal et al. estimated that the presence of CHIP conferred a 0.5% per year risk for developing a hematological neoplasm, which increased to 1% per year if the VAF exceeded 10% [34]. Similarly, Cordua et al. reported a 14% increase in the risk of MPN for each additional percentage of VAF [39].

In many individuals with MPN, *JAK2* p.V617F is the sole somatic mutation detected [42,56]. In a 2017 study involving nine MPN patients with the *JAK2* p.V617F variant who had previously donated blood, wide variability between individuals whose clones only harbored *JAK2* p.V617F was shown, with a clonal proliferation rate that ranged from 0.36% to 6.2% per year, resulting in latencies (defined as the time between variant detection and MPN diagnosis) of 4.6 to 15.2 years [57]. Single-cell transcriptional studies using RNA-seq have also revealed that the fraction of *JAK2*-mutated HSPCs is variable among patients, as is the affectation of the progenitor cells: Megakaryocytic and erythrocytic progenitors tend to have the highest VAF, while lymphoid progenitors have the lowest VAF [58,59,60].

## 4. Latency

Studies on latency in patients with MPN face various challenges, such as the absence of serial sampling spanning several decades and clonal heterogeneity, i.e., the existence of multiple clones with different fitness that are indistinguishable by the sequencing of PB or total BM. However, in recent years, advances in single-cell sequencing techniques have permitted the investigation of clonal evolution via the construction of phylogenetic trees of tumoral clones using a method known as retrograde extrapolation [58,61,62].

These phylogenetic trees are utilized to analyze neoplastic clones and trace their evolutionary paths, in a similar way to how evolutionary relationships between different species are demonstrated. By examining serial samples from the same patient, researchers can determine the trajectory of clonal expansion and estimate the latency period between the acquisition of the mutation and the clinical detection of MPN. Through the application of these techniques, pioneering studies have inferred that the *JAK2* p.V617F mutation can be acquired various decades before MPN diagnosis [58,62]. For example, one study found that in cases where *JAK2* p.V617F was the sole driver mutation, the average latency period was 34 years [62]. 

Another new technique used to estimate latency is the passenger-approximated clonal expansion rate (PACER). PACER allows the prediction of the clonal expansion rate using data from a single sample by considering the total number of passenger mutations and adjusting for the VAF and the age of the individual [63]. This technique is based on the principle that as a mutant clone expands, the VAF of both driver and passenger mutations increases. Thus, for two individuals of the same age, the clone with more passengers would be expected to be fitter since it would have expanded to the same size in less time. 

Groundbreaking retrograde extrapolation studies have provided insight into the acquisition of somatic mutations in a very early stage of development, even in utero [58,62]. These results, although controversial, have been supported by other observational findings, such as the detection of the same mutation in *SRSF2* or *DNMT3A* in two pairs of twins with MPN [64,65]. Furthermore, a study published in Nature Medicine in 2022 reported the presence of a somatic mutation in *CALR* in two twins who developed MF at the ages of 37 and 38. The mutation was shown to have originated in a hematopoietic stem cell (HSC) in utero as a result of transplacental transmission to the twins. The same study also demonstrated the in utero origin of *JAK2* p.V617F, which was detected with a VAF of 1% in a blood drop collected at birth in the “heel prick” neonatal screen. The individual carrying this mutation was later diagnosed with PV at the age of 34 [65].

## 5. Clonal Expansion

The early acquisition of CHIP mutations and/or MPN drivers, such as *CALR*, without conferring a clonal advantage, suggests that these mutations may be present decades before the onset of MPN. During this period, these mutations may not significantly impact the fitness of HSCs compared to other HSCs in the BM. However, while the VAF of *JAK2* can remain stable in some patients over several years [66], the size of the clone and the number of mutations generally increase with age. 

Hence, it is evident that other biological factors must influence clonal expansion and the progression of a clone harboring *JAK2* p.V617F (or other CHIP mutations or chromosomal alterations) to a symptomatic MPN clone.

### 5.1. Other Mutations

One contributing factor is the mutational burden. The presence of additional mutations or multiple chromosomal alterations can have an impact on the rate of clonal expansion. Thus, clones with a higher number of mutations have a higher risk of progressing to a hematological neoplasm. 

Additionally, mutations in different genes have been shown to induce different rates of clonal growth or expansion. A study that followed 697 CHIP clones from 385 individuals over a median period of 13 years found that although most clones expanded at a stable exponential rate, the presence of additional mutations could accelerate the rate of clonal expansion [57]. The authors observed that certain mutations affected the growth rate differently, from 5% for mutations in *DNMT3A* and *TP53* to 50% per year in the case of *SRSF2* p.P95H. Importantly, the study also found that the presence of the *SRSF2* p.P95H mutation was associated with a higher risk of progression to neoplasm, highlighting the clinical significance of different clonal expansion rates [67].

In a seminal study, it was observed that the specific gene mutation and the order in which mutations in different genes are acquired could both have an impact on the age at which MPN manifests [68]. Specifically, in MPN patients with mutations in both *JAK2* and *TET2*, the acquisition of *TET2* mutation prior to *JAK2* mutation was found to diminish the proliferative effect of *JAK2* p.V617F, resulting in an earlier manifestation of MPN and tendency towards an ET phenotype, compared to patients where the *JAK2* mutation occurred first with a tendency towards a PV phenotype. Similarly, when 10 MPN patients all harboring the *JAK2* p.V617F variant were studied by retrograde extrapolation, the development of a *JAK2* mutation first was associated with an increased propensity toward PV or MF, whereas the development of a *DNMT3A* mutation first was associated with an increased propensity toward ET [69].

### 5.2. Germline Predisposition

The risk of developing MPN has been demonstrated to be five to seven times higher for individuals who have a first-degree relative affected by MPN [70], and numerous germline variants and polymorphisms have been described that increase the predisposition to MPN. For instance, carriers of the rs34002450 germline variant of *TERT* (which encodes telomere enzyme reverse transcriptase) have a significantly increased risk of developing both CHIP and MPN [31,71].

Other germline mutations are specifically associated with HSPC self-renewal. For example, the *JAK2* 46/1 haplotype or GGCC (rs1327494) confers a two to six times increased risk of developing MPN or CHIP [72,73,74]. 

Indeed, in a genome-wide association study (GWAS) with the aim of identifying genes whose mutation is associated with an increased MPN risk, 11 of the 15 genes identified included *JAK2*, *SH2B3* (sometimes referred to as *LNK*), *TET2*, *RUNX1*, *ATM*, and *TERT*, as well as other factors with a role known to be associated with HSC self-renewal and function, such as *GATA2*, *HMGA1*, *FOXO1*, and *MECOM* [75].

Specifically, there is a positive correlation between inherited genetic risk for MPN and CHIP ([76], Table 1). One GWAS study of germline variants associated with CHIP observed that individuals with the A allele at SNP rs79901204 had a 2.4-fold increased risk for CHIP due to the disruption of a *TET2* enhancer, resulting in decreased TET2 expression [71].

Moreover, a study of germline mutations in 5071 individuals with CHIP used PACER to identify a novel locus within the *TCL1A* promoter (rs2887399), which was shown to be associated with slower clonal expansion of CHIP [63]. This variant specifically increased the risk of *DNMT3A* CHIP and mosaic loss of chromosome Y but decreased the risk of acquiring *TET2* CHIP mutations. Additionally, analysis of whole exome sequencing data from over 200,000 UK Biobank participants identified 14 germline associations with CHIP in European populations [75]. These associations included variants in genes such as *CHEK2*, *PARP1*, *ATM*, *CD164*, and *SETBP1*. Thus, it is noteworthy that many known germline risk factors for myeloid diseases may also contribute to the clonal expansion that leads to CHIP, given their role as a precursor state to hematological neoplasms.

Furthermore, germline mutations in genes associated with genome stability, such as *CHEK2* (Table 1), lead to an increased susceptibility to acquiring additional mutations. Germline mutations in *CHEK2* have significant implications for stem cell self-renewal [76,78] and elevate the risk of tumor development in general. Through GWAS studies, numerous germline mutations that predispose individuals to CHIP have been identified (reviewed in [77]). Among them are germline variants in *KPNA4* and *MBD4*, the latter of which plays a protective role against methylation damage. Variants in these genes are associated with an increased risk of *JAK2*-mutated CHIP and early-onset AML, respectively [71,81]. 

### 5.3. Inflammation

There is ever-increasing evidence highlighting the important role that chronic inflammation plays in the pathogenesis of MPN. Chronic inflammation triggers the activation of inflammatory pathways, leading to an increased production of cytokines, which confers a competitive advantage to the disease clone [83,84,85]. A population study involving 11,039 MPN patients and 43,550 controls demonstrated a significantly higher risk of developing MPN among individuals with an autoimmune disease, including Crohn’s disease and Reiter’s syndrome [86]. Furthermore, chronic inflammation can cause genomic instability through the generation of reactive oxygen species (ROS), resulting in DNA damage. This DNA damage contributes to the progression of the disease and further enhances the clonal expansion and survival of MPN cells [85,87]. 

Proinflammatory cytokines have been suggested to promote the expansion of *DNMT3A*-mutated clones [88]. Similarly, it has been observed that monocytes in MPN patients exhibit reduced sensitivity to the anti-inflammatory cytokine IL-10, resulting in the continuous production of TNF-alpha [89]. It has been proposed that this inflammatory stimulus could provide *JAK2* p.V617F cells with a competitive advantage. In this respect, it is suggested that an individual’s genome could be influenced by their predisposition to inflammation [90]. Supporting this idea, a study found a reduction in IL-10R signaling in the twin of an MPN patient, even though the twin did not have MPN. This finding suggests that the signaling anomaly was acquired prior to the development of MPN. A second example of how genetic factors influence inflammatory pathways and disease predisposition is the observation that individuals with CHIP and a loss-of-function polymorphism in the *IL6R* gene have a reduced risk of cardiovascular disease and development of MPN [91].

Another source of inflammation includes the microbial production of harmful molecules in the gut, such as TLR2 agonists released by several *Lactobacillus* strains, which can enter into the bloodstream and have been shown to induce IL-6 production resulting in increased myeloproliferation in Tet2^−/−^ mice [92,93]. Ongoing studies are continuing to explore the impact of inflammation on the development of MPN. These studies include the analysis of the role of the intestinal microbiome in MPN [94] and the NUTRIENT clinical trial (NCT03907436), which aims to evaluate the effectiveness of an anti-inflammatory diet in MPN patients [95].

To summarize, the acquisition of additional factors or mutations (such as *DNMT3A* mutations), exposure to cell-extrinsic factors (such as UV light and genotoxic agents), or intrinsic cellular factors (like inflammation or aging), can lead to an increase in clonal fitness and the subsequent development of MPN. Specifically, age-related changes, both biochemical and functional, have been observed to affect HSC fitness [96,97]. For example, retrograde extrapolation experiments analyzing blood DNA samples from 385 older individuals revealed a rapid decline in clonal diversity in those over 60 years, revealing oligoclonal CHIP [67]. Moreover, individuals with *TET2*-mediated CHIP showed the greatest “epigenetic age acceleration”, a phenomenon where an individual’s biological age, as measured by epigenetic markers such as age-related methylation, appears to be progressing at a faster rate than their chronological age [98,99]. Nevertheless, in many cases, the clonal expansions in older individuals lacked recognizable drivers [67,77,100]. 

This reduced fitness associated with older age and selection for mutations that confer a clonal advantage can promote MPN development, and indeed leukemic transformation, as well as provide a more permissive environment for clonal expansion, i.e., due to chronic inflammation [67,101].

## 6. Can Early-Phase MPN Be Detected in the Clinic?

### 6.1. Screening Feasibility

Two studies have assessed the feasibility of screening healthy individuals for the presence of *JAK2* mutations [102,103]. In the first study, a two-step algorithm was developed to screen individuals suspected of having PV [102]. The study analyzed 15,366 peripheral blood samples, focusing on those showing elevated levels of hemoglobin or hematocrit according to WHO criteria [104]. Of the 996 individuals who met the screening criteria, eight individuals (0.8%) tested positive for *JAK2* p.V617F mutation (considering a VAF above 0.71%). The study also found that individuals with *JAK2* p.V617F mutation had significantly higher levels of neutrophils and platelets and established cut-off values of 6 × 10^9^/L and 250 × 10^9^/L, respectively. When this algorithm was applied to an independent validation cohort, *JAK2* p.V617F was detected in 1.2% of individuals [102].

In the second study, a mutation screen was conducted using multiplex digital PCR to detect *JAK2* p.V617F and *CALR* mutations (using a threshold of VAF ≥ 1%) in both healthy individuals and individuals with elevated levels of hemoglobin, hematocrit, leukocytes, or platelets in two consecutive blood tests [18]. Among the 373 individuals with high blood counts, 32 (8.5%) tested positive for *JAK2* (none tested positive for *CALR* mutation), with an average VAF of 1.3%. Among the 19958 healthy individuals tested, 3.2% were mutated (613 in *JAK2*, 32 in *CALR*), with an average VAF of 2.4%. Further analysis revealed that 12 individuals with mutations and 10 individuals without mutations had BM findings compatible with MPN. Although three out of 25 individuals (12%) who tested positive for mutations also had elevated blood counts in consecutive hemograms, the authors of the study suggested that the mutational screening method was more sensitive, given that five out of 12 individuals (42%) with early-phase MPN would not have been detected by the method of elevated blood counts alone [18]. Finally, using a Receiver Operating Characteristic (ROC) curve, the authors established that a VAF threshold of 1% had a predictive value of 79% for detecting changes in the BM biopsy. 

Both of these studies conclude that the detection of *JAK2* p.V617F at low VAF in healthy individuals could be a feasible method for the early diagnosis of MPN. However, prospective clinical studies are required to determine the value of monitoring individuals with a low VAF, establish the threshold of “low VAF”, and decide the appropriate frequency for repeating the determination. Furthermore, it remains to be determined whether individuals with a high VAF of mutated *JAK2* and/or other mutations (such as *SRSF2* p.P95H) would benefit from a closer follow-up. However, it is worth mentioning here that other researchers observed that abnormal blood counts were associated with a higher risk of developing a myeloid neoplasm, even in patients without genetic alterations [32].

An alternative method for monitoring early-phase MPN patients could involve assessing the fitness of MPN clones as a prognostic biomarker. One study monitored *JAK2*-mutated MPN clone fitness by measuring the VAF of *JAK2* p.V617F with digital PCR in 11 distinct cell populations [59]. Unsupervised hierarchical clustering of *JAK2* p.V617F VAF within the 11 populations identified four major MPN fitness levels. Patients in higher fitness groups were more likely to experience adverse events, such as thrombosis, hemorrhage, disease progression, or death, whereas VAF of whole blood was not predictive, indicating its inadequacy as a monitoring biomarker.

### 6.2. Possible Intervention

During the initial phase, there is an opportunity to implement preventive measures and interventions aimed at delaying or preventing the onset of symptomatic disease and its associated thrombotic complications. One preventive strategy could involve an annual hemogram to molecularly monitor individuals with an increased VAF greater than 1% (or another yet-to-be-defined threshold) [104]. Although MPN evolution from *JAK2* p.V617F CHIP is not certain, a *JAK2* p.V617F VAF greater than 2% was associated with a high incidence of CHIP to MPN progression, as was a demonstrated increase in VAF during follow-up [18,60,105]. Similarly, monitoring of M-CHIP could be of interest to national health systems in terms of prevention since it is associated with cardiovascular disease, higher mortality, and an increased risk of developing a myeloid neoplasm [34]. In the screening conducted by Piris et al., one-third of the patients with *JAK2* p.V617F later experienced a cardiovascular event [102]. Considering the increasing age of the population, this consideration gains importance as the incidence of both MPN and CHIP is expected to rise. 

A simple intervention to prevent MPN-associated thrombosis could be early prophylaxis with aspirin. However, the ASPirin in Reducing Events in the Elderly with Clonal Hematopoiesis trial (ASPREE CHIP) showed that low-dose aspirin had no effect on reducing the risk of cardiovascular events in individuals with CHIP [106].

Ongoing research is exploring different interventions to reduce chronic inflammation. Some promising results have been achieved with antioxidant therapy. For example, studies conducted on mice with a BM graft of *JAK2* p.V617F cells have shown that supplementation with the antioxidant N-acetylcysteine can lead to a decrease in DNA damage, reduced splenomegaly, and fewer thrombotic complications [87,107]. Moreover, the dietary supplementation of MPN patients with N-acetylcysteine led to improvements in the symptomatic burden [108]. Additionally, vitamin C (ascorbic acid) levels in HSCs have been found to be 2- to 20-fold higher compared to other hematopoietic progenitors. Vitamin C acts as a cofactor for TET protein-mediated DNA demethylation functions [109,110]. Indeed, low levels of vitamin C in HSCs can mimic TET2 loss of function [109,110], leading to increased HSC frequency. On the other hand, ultra-high vitamin C doses reverted leukemia progression in patient-derived xenograft models [111]. Notably, vitamin C was found to delay cancer growth via T cell-dependent infiltration of the tumor microenvironment, but only when given intravenously at high doses [112]. Indeed, an ongoing clinical trial of patients with low-risk *TET2*-mutated myeloid malignancies, including clonal cytopenia of undetermined significance (CCUS), low-risk MDS, and CMML, is evaluating whether intravenous vitamin C can reduce the VAF and/or number of mutations (NCT 03682029). 

Other strategies to reduce inflammation could include interferon-alpha (IFN-α) treatment, which was shown to reduce the size of the *JAK2* p.V617F clone in MPN patients [113,114,115] by pushing the mutant HSCs into the cell cycle and exhausting them. Type I IFN may even inhibit IL-1β production [116]. Thus, one interesting therapy to prevent cardiovascular events for MPN patients with CHIP could be canakinumab, an anti-interleukin-1β monoclonal antibody. Patients enrolled in the Canakinumab Anti-Inflammatory Thrombosis Outcome Study (CANTOS) study with *TET2* mutations responded better to canakinumab than patients without CHIP-associated mutations [117]. Moreover, loss of interleukin-1β (IL-1β) decreased the frequency of MPN disease initiation in mouse models of MPN [118]. Meanwhile, for *JAK2*-mutated CHIP, an inhibitor of IL-18 may be a more appropriate strategy, since *JAK2*-mutated CHIP is associated with increased levels of IL-18 and IL-6 rather than IL-1β [71]. Thus, a potential intervention may need to be tailored according to the driver mutation and/or CHIP presented by the individual. 

Before any possible screening program could be launched, the appropriate infrastructure must be in place, including sufficient personnel and equipment. To this end, the development and introduction of a large-scale *JAK2* p.V617F detection system would facilitate potential *JAK2* mutation screening in the general population [102]. A more cost-efficient alternative could be to selectively screen individuals at high risk of developing an MPN. Examples of such high-risk groups include the relatives of a patient with a confirmed MPN diagnosis [70,119], smokers [120,121], or patients with an autoimmune disease [86]. 

Finally, the introduction of a *JAK2* p.V617F screening program should only go ahead after a thorough evaluation of the advantages and disadvantages associated with such screening. Possible disadvantages to consider include insufficient resources to effectively monitor individuals with a mutation detected and the anxiety that may arise in patients who receive a “positive” result [16]. Furthermore, the preventative treatment of individuals with initial-phase MPN should certainly not include the unnecessary treatment of individuals who may never develop overt MPN during their lifetime. It is worth noting that, to date, no intervention has been shown to delay the onset of MPN effectively. 

## 7. Conclusions and Future Perspectives

With the current technologies available in hematology laboratories, such as quantitative PCR or digital PCR, it is possible to detect driver mutations (and/or CHIP mutations) with a low VAF in the early phase of a disease, even several decades before the onset of a hematological malignancy. These mutations can be acquired in childhood, or even in utero, in some individuals, and can be detected in stored neonatal screening samples taken several decades ago. Findings from the numerous molecular studies presented in this review highlight the potential for somatic mutations to arise early in development and contribute to the subsequent development of hematological disorders such as MPN after clonal expansion over several decades. 

**Figure 1:**
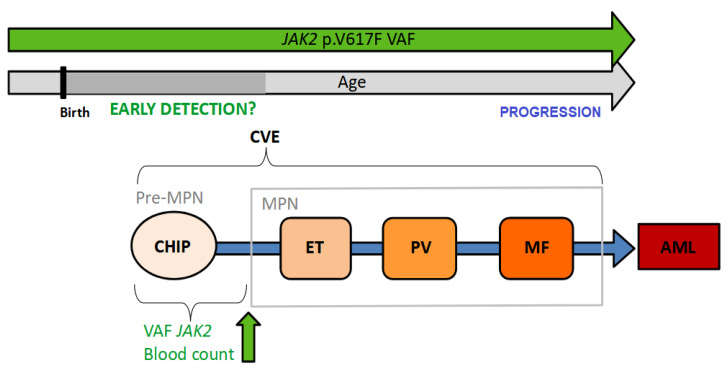
The biological continuum from the development of CHIP or “pre-MPN” to the manifestation of an overt MPN opens opportunities for early diagnosis and intervention. The asymptomatic initial phase can last for several decades, during which the *JAK2* p.V617F variant can be detected, from birth in some cases. AML: acute myeloid leukemia; CHIP: clonal hematopoiesis of indeterminate potential; CVE: cardiovascular events; ET: essential thrombocythemia; MF: myelofibrosis; PV: polycythemia vera; VAF: variant allele frequency.

Although the presence of *JAK2* mutations in the “healthy” population complicates interpretation, several studies have demonstrated the feasibility of the early identification of MPN patients via *JAK2* p.V617F screening in asymptomatic individuals, even before changes in the blood count are detectable [19,103]. Perhaps it is time to change our definition of a “healthy” individual to a “pre-MPN” in the case of individuals with a low VAF *JAK2* mutation [40] (Figure 1).

Nevertheless, this screening strategy would not be suitable for capturing all MPN patients, since some 10–15% of patients with MF or ET are triple negative and have as yet undiscovered drivers of their disease [122]. Moreover, no association between *CALR* mutations and CHIP has been described to date, although patients with *CALR*-mutated ET are diagnosed at a significantly younger age, suggesting a higher proliferative advantage of the *CALR*-mutated HSC compared to a *JAK2*-mutated HSC [123]. Moreover, *CALR*-mutated MPNs are known to have an increased risk of myelofibrotic transformation and reduced risk of thrombosis, particularly for the *CALR* type-2 mutation [124,125,126]. 

There are still unresolved questions regarding the development of MPN, such as why there is so much variability in the latency period between individuals with the same driver mutation. Moreover, while studies on the sequence of molecular events are helping to elucidate factors associated with clonal expansion, the biological factors that contribute to the transformation of an asymptomatic *JAK2*-mutated clone into an overt MPN clone remain to be determined. The identification of other biomarkers associated with the progression from CHIP to overt MPN could also facilitate the early detection of these neoplasms and provide insights into potential interventions. What is apparent from studies conducted to date is that there is a window of opportunity stretching over several decades for a potential intervention to be implemented. 

An ideal intervention would slow the clonal expansion of *JAK2*-mutated clones (and/or CHIP) and thus delay or prevent the development of overt MPN and associated comorbidities. Possible interventions currently under consideration include dietary supplementation with antioxidants or early treatment with immunomodulatory agents like interferon [19]. However, at this time, the only feasible interventions would include the monitoring of blood counts and strategies to reduce associated vascular risk, such as lifestyle changes and prophylaxis with low-dose aspirin [127]. 

In the future, artificial intelligence (AI) could potentially play a significant role in the early detection of MPN due to its predictive modeling capacity. Machine learning can process vast amounts of medical data, including electronic health records, medical images (such as bone marrow biopsies or blood smears), laboratory results, and even genomic data to predict the likelihood of an individual developing MPN based on various risk factors and early symptoms. For example, Sirinukunwattana et al. created an automated workflow based on histological features, such as the characteristics of megakaryocytes, to diagnose MPN from BM biopsies [128]. Additionally, Mosquera et al. applied machine learning techniques to a large dataset of clinical data from patients with MF, developing a predictive model for risk stratification [129].

In conclusion, this article has highlighted that there is still much work to be carried out in understanding why some clones expand into a malignant MPN clone during a lifetime and others do not. Such knowledge would help develop intervention opportunities to minimize the risk of complications associated with MPN (including thrombosis, hemorrhages, or MPN progression to more aggressive phases) and thus reduce a huge economic burden for national health systems.

## Figures and Tables

**Table 1:** Single-nucleotide polymorphisms (SNP) associated with an increased risk of developing clonal hematopoiesis of indeterminate potential (CHIP). Adapted from [77]. The Single Nucleotide Polymorphism database (dbSNP) annotation is indicated for each variant.

Gene	Function	dbSNP	Effect	Reference
*JAK2* 46/1 haplotype	Key driver mutation in MPN.	rs1327494	Higher risk of acquiring *JAK2* p.V617F-mutated CHIP or MPN.	[71,72,73,74]
*TET2*	Role in myelopoiesis. Loss of this gene has been associated with MPN and CHIP.	rs144418061	Variant specific to African ancestry in an intergenic region near *TET2.* Carriers of the A allele have a 2.4-fold increased risk of CHIP.	[71]
		rs1548483	SNP upstream of *TET2* associated with *JAK2*-mutated CHIP and MPN.	[75]
		rs79901204	Disrupts a *TET2* distal enhancer resulting in decreased *TET2* expression, increased HSC self-renewal, and increased risk of acquiring any CHIP driver mutation.	[71]
*TERT*	Telomere enzyme reverse transcriptase. Key role in telomere length maintenance.	rs7705526	Frequent variant in the 5th intron of *TERT* associated with CHIP, and specifically with *JAK2*-mutated CHIP and MPN.	[71,75,78]
		rs34002450	The A allele confers a 1.3-fold increased risk of developing CHIP.	[71,79]
		rs2853677	Identified in European populations.	[78]
		rs13156167 E2D;rs2086132	Associated specifically with *DNMT3A*-mutated CHIP.	[76]
		rs2736100	Associated with *TET2*-mutated CHIP and MPN.	[76]
*CHEK2*	DNA damage repair.	rs555607708 E2D;	Higher risk of being a *JAK2* p.V617F carrier and developing multiple mCAs.	[31,75]
		rs62237617	The T allele conferred a large increase in the risk of *DNMT3A*-mutated CHIP.	[78]
*ATM*	DNA damage repair.	rs1800056	Higher risk of being a *JAK2* p.V617F carrier.	[75]
		rs11212666	Associated specifically with *DNMT3A*-mutated CHIP in a European population.	[78]
*SH2B3*	Mutations identified in MPN and result in aberrant JAK-STAT signaling.	rs7310615	Associated with *JAK2*-mutated CHIP and MPN.	[75]
*GFI1B*	Transcriptional repressor with key role in hematopoiesis.	rs524137	SNP in the enhancer region resulted in 2.7-fold increase in the expansion of HSC.	[80]
		rs621940	Higher risk of being a *JAK2* p.V617F carrier.	[75]
*SMC4*	Encodes condensin subunit with key role in chromosome segregation.	rs12632224	Identified in European populations.	[78]
*PARP1*	DNA damage repair.	rs138994074	Associated specifically with *DNMT3A*-mutated CHIP in a European population.	[78]
*CD164*	HSC migration/homing.	rs35452836	Identified in European populations.	[78]
*ENPP6*	Enzyme with a role in choline metabolism.	rs13130545	Identified in European populations.	[78]
*SETBP1*	Myeloid oncogenesis.	rs8088824	Associated specifically with *DNMT3A*-mutated CHIP in a European population.	[78]
*MBD4*	Role in DNA mismatch repair.	rs79901204	Individuals with the A allele have a 2.4-fold increased risk for CHIP due to the disruption of a *TET2* enhancer resulting in decreased *TET2* expression. Particularly prone to *DNMT3A*-mutated CHIP.	[81]
*TCL1A*	Factor that enhances cell proliferation.	rs2887399	Carriers of the T allele have 1.2-fold increased risk of CHIP, particularly *DNMT3A*-mutated CHIP in the European population. The variant is also associated with mosaic loss of chromosome Y.	[71,78,82]
		rs10131341	Associated specifically with *TET2*-mutated CHIP in European population.	[78]
*KPNA4-TRIM59* locus	Role in nuclear protein import.	rs1210060191	Carriers of this common variant (1-base pair deletion) have a 1.2-fold increased risk of CHIP, including *JAK2*-mutated CHIP.	[71]
*PINT*	No known role in HSC biology.	rs58270997	Higher risk of being a *JAK2* p.V617F carrier.	[75]
*TMEM209*	Integral protein of nuclear envelope.	rs79633204	Associated specifically with *TET2*-mutated CHIP in a European population.	[78]

CHIP: clonal hematopoiesis of indeterminate potential; HSC: hematopoietic stem cells; mCA: mosaic chromosomal alteration; MPN: myeloproliferative neoplasm; SNP: single nucleotide polymorphism.

## Data Availability

Not applicable.
